# Effect of Physical Activity, Smoking, and Sleep on Telomere Length: A Systematic Review of Observational and Intervention Studies

**DOI:** 10.3390/jcm11010076

**Published:** 2021-12-24

**Authors:** Rocío Barragán, Carolina Ortega-Azorín, Jose V. Sorlí, Eva M. Asensio, Oscar Coltell, Marie-Pierre St-Onge, Olga Portolés, Dolores Corella

**Affiliations:** 1 Department of Preventive Medicine and Public Health, School of Medicine, University of Valencia, 46010 Valencia, Spain; rocio.barragan@uv.es (R.B.); carolina.ortega@uv.es (C.O.-A.); sorli@uv.es (J.V.S.); eva.m.asensio@uv.es (E.M.A.); olga.portoles@uv.es (O.P.); 2 CIBER Fisiopatología de la Obesidad y Nutrición, Instituto de Salud Carlos III, 28029 Madrid, Spain; oscar.coltell@uji.es; 3 Sleep Center of Excellence, Department of Medicine, Columbia University Irving Medical Center, New York, NY 10032, USA; ms2554@cumc.columbia.edu; 4 Division of General Medicine, Department of Medicine, Columbia University Irving Medical Center, New York, NY 10032, USA; 5 Department of Computer Languages and Systems, Universitat Jaume I, 12071 Castellon, Spain

**Keywords:** aging, telomere length, physical activity, exercise, tobacco, smoking, sleep, lifestyle

## Abstract

Aging is a risk factor for several pathologies, restricting one’s health span, and promoting chronic diseases (e.g., cardiovascular and neurodegenerative diseases), as well as cancer. Telomeres are regions of repetitive DNA located at chromosomal ends. Telomere length has been inversely associated with chronological age and has been considered, for a long time, a good biomarker of aging. Several lifestyle factors have been linked with telomere shortening or maintenance. However, the consistency of results is hampered by some methodological issues, including study design, sample size, measurement approaches, and population characteristics, among others. Therefore, we aimed to systematically review the current literature on the effects of three relevant lifestyle factors on telomere length in human adults: physical activity, smoking, and sleep. We conducted a qualitative systematic review of observational and intervention studies using the Preferred Reporting Item for Systematic Reviews and Meta-Analysis (PRISMA) guidelines. The systematic literature search covered articles published in MEDLINE and EMBASE databases (from 2010 to 2020). A total of 1400 studies were identified; 83 were included after quality control. Although fewer sedentary activities, optimal sleep habits, and non- or ex-smoker status have been associated with less telomere shortening, several methodological issues were detected, including the need for more targeted interventions and standardized protocols to better understand how physical activity and sleep can impact telomere length and aging. We discuss the main findings and current limitations to gain more insights into the influence of these lifestyle factors on the healthy aging process.

## 1. Introduction

Life expectancy has increased in recent decades, and it is expected to rise in the coming years [1,2,3,4]. Therefore, there is a higher probability of suffering from diseases related to an aging population, such as cardiovascular diseases [1,5,6,7], diabetes [7,8,9], hypertension [10,11,12], cancer [7,13], and neurological diseases [7,14]. Owing to the social, psychological, and economic conditions associated with aging, this has now become one of the major issues facing public health [15,16,17].

Aging is defined as a diminishing of biological functions over time, in which there is a progressive loss of the function and structure of tissues and systems [6,18]. The underlying mechanisms of the this process have been studied in depth and it seems they are related with genetic and epigenetic alterations, proteostasis loss, telomere shortening, autophagy deterioration, and mitochondrial dysfunction, among others [1,4,18,19]. However, the specific role of each mechanism remains unknown. Furthermore, these mechanisms may be modulated by other risk factors, such as diet, tobacco smoking, physical activity (PA), stress, or sleep, which, in turn, have been associated with certain diseases related to aging [18]. Therefore, a better knowledge of effective strategies for slowing the aging process is an important priority for researchers and policy makers.

Currently, several biomarkers of biological aging have been developed. Telomeres are regions of repetitive sequences (TTAGGG)_n_ and associated protective proteins located at the ends of the cap-shaped linear chromosomes that preserve the integrity and stability of chromosomes during DNA replication. They are essential to chromosome stability and protect genomic DNA through various mechanisms [20,21,22,23,24]. Usually, a relative quantification protocol by calculating the telomere/single copy gene ratio is undertaken.

The telomere is stabilized by a six-protein complex called “shelterin”, including the TRF (telomeric repeat binding factor)1, TRF2, POT1 (protection of telomeres 1), TINF2 (TERF1-interacting nuclear factor 2), RAP1 (repressor activator protein 1 and TPP1 (tripeptidyl peptidase 1) [21,22]. Telomeres are subject to shortening during each cell division as the DNA replication mechanism cannot copy the DNA in its entirely. The enzyme telomerase, however, can solve this problem by adding the repetitive sequences and maintaining telomere length (TL). Telomere shortening is one of the most frequently studied mechanisms in the aging process. Telomere attrition reflects the cumulative load of oxidative, inflammatory, and mechanical stress [18,25]. Hence, TL has been proposed as a biomarker of biological age and a risk factor for chronic diseases [4,18,25,26,27,28,29,30] and total mortality [22,25,28,29,31,32]. Nevertheless, the association between these outcomes and the TL marker are still under discussion due to inconsistent results [19,25,33,34,35,36,37,38,39,40].

One of the factors potentially contributing to the heterogeneous results is the measurement approach used to determine TL. This is an important issue given the fact that TL differs in leukocyte subtypes isolated from the same individual. Although blood leukocytes are the most commonly used samples to determine TL, it is known that TL may substantially vary across various tissue types. The terminal restriction fragment (TRF) approach is the oldest method for analyzing TL and remains the “gold standard” even in current studies. It uses the southern blot or in-gel hybridization with a specific proof for telomere DNA [17,18]. Other methods include the fluorescent in situ hybridization (FISH) by using digital microscopy (quantitative FISH as Q-FISH) and flow cytometry (Flow-FISH). Another method, telomere shortest length assay (TeSLA), measures the distribution of shortest telomeres in heterogeneous situations. It provides a better measurement of the shortest length of telomeres. However, the quantitative polymerase chain reaction (PCR) is most widely used for its high throughput. Despite the fast, highly sensitive nature of PCR approach, which requires low amounts of DNA, the distribution of long and short telomeres cannot be obtained, and it is not possible to evaluate TL of individual cells or chromosomes [17,18]. This method is often chosen for large cohort studies, using DNA from leukocytes. Additionally, telomerase activity, gene expression, or epigenetic regulation of telomere-related genes are analyzed to delve deeper on the mechanisms [20,21,24].

TL is determined by the interaction between endogenous and external factors that explain greater or lesser susceptibility to accelerated aging [22]. Therefore, the exposome could well play a fundamental role through various biological mechanisms in the maintenance of the telomeres during aging [41,42]. Several studies have analyzed the association between lifestyle and TL, both as a protective factor [16,43,44], and as inducer of their shortening [36,45,46]. Diet has been one of the most commonly studied lifestyle factors with regard to TL. Several studies have shown the benefit of consuming foods rich in antioxidants or the benefits of various diets, such as Mediterranean type diets, on telomere attrition, as witnessed in a number of the already published reviews [47,48,49,50,51,52]. However, other relevant lifestyle factors related to aging and chronic diseases [53,54,55,56,57,58,59], such as PA, smoking or sleep have not been examined in such depth. Therefore, the aim of this work is to conduct a systematic review of the current literature (including observational and intervention studies) on the effects of PA, smoking, and sleep, on TL in relatively healthy human adults.

## 2. Materials and Methods

### 2.1. Search Strategy

We conducted a literature search for studies investigating the relationship between PA, smoking and sleep on TL in adults. In line with established guidelines (PRISMA) (http://www.prisma-statement.org/; accessed on 10 November 2021), a systematic review was carried out in which studies from the MEDLINE and EMBASE databases have been included. This was supplemented by a manual search of articles published from 2010 to March 2020. The search strategy for each lifestyle factor was made independently. The search terms employed were as follows: (“Physical Activity” OR “Exercise” OR “Smoking” OR “Tobacco” OR “Sleep”) AND (“Telomere”). The protocol for the systematic review is available at PROSPERO, the NIHR International prospective register of systematic reviews (https://www.crd.york.ac.uk/PROSPERO/; accessed on 10 November 2021). Registration number: CRD42020192881.

### 2.2. Inclusion and Exclusion Criteria

Bibliographical inclusion was restricted to observational (cross-sectional, longitudinal studies, case-control studies and cohorts) and experimental studies (interventions) from original articles and carried out on healthy adults of both sexes, different ethnic backgrounds or with overweight, obesity or diabetes as these conditions (mainly overweight and obesity) are prevalent in studies focusing on the general population. Excluded were studies in adolescents, children, pregnant women, individuals with infectious diseases, and other chronic pathologies, or individuals consuming addictive substances, or with respiratory disorders or chronic sleep problems. Reviews, meta-analyses, conference summaries, unpublished studies, and studies published in languages other than English were also excluded from this systematic review.

### 2.3. Quality Control Analysis

The selection and assessment of the quality of the articles included herein were performed independently by three reviewers (R.B., C.O.-A., and J.V.S.). The quality of each study was determined by the tools provided by the National Heart, Lung, and Blood Institute (NHLBI) of the National Institutes of Health (NIH) for evaluating the quality of observational and experimental studies. Once a score was obtained, the studies were grouped by quality, as good, acceptable, or low quality. Following this appraisal, 78% of the studies included in this review were classified as good quality.

## 3. Results

**Figure 1:**
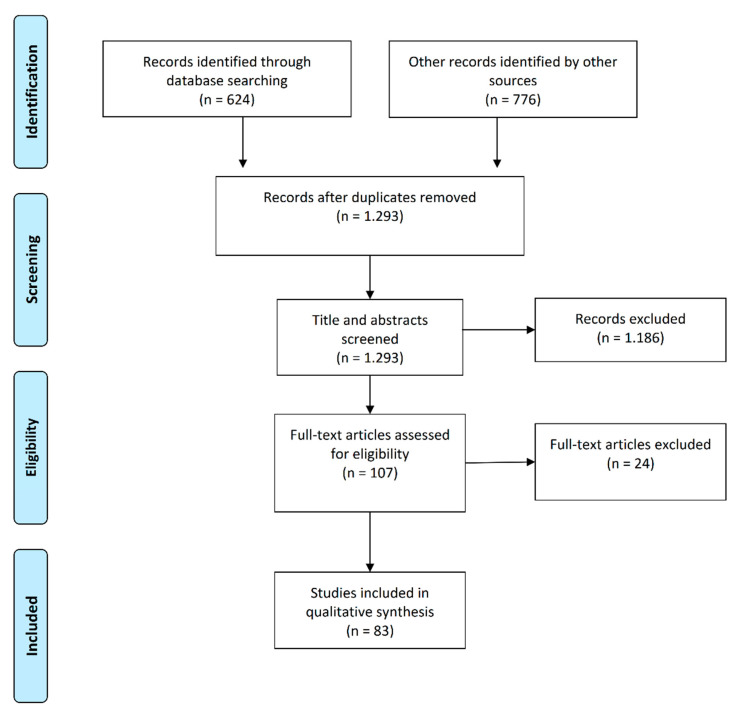
Workflow of the systematic review according to PRISMA 2009 flow chart.

In the initial search (Figure 1), 1400 articles were identified, of which 83 were included. The detailed characteristics of these studies are presented in Table 1 [45,46,60,61,62,63,64,65,66,67,68,69,70,71,72,73,74,75,76,77,78,79,80], Table 2 [27,81,82,83,84,85,86,87,88,89,90,91,92,93], Table 3 [44,94,95,96,97,98,99,100,101,102,103,104], Table 4 [60,67,77,93,105,106,107,108,109,110,111,112,113,114,115,116,117,118,119,120], Table 5 [121,122,123,124,125,126,127], and Table 6 [43,128,129,130,131,132,133,134,135,136,137]. Most studies focused on the association between TL and PA (Table 1, Table 2 and Table 3), followed by smoking (Table 4 and Table 5), and sleep (Table 6). Four articles were included in more than one table as in those papers, both PA and smoking were analyzed separately, and the corresponding results were included in the respective Tables.

### 3.1. Association between PA and TL

The impact of PA as a relevant factor in preventing diseases associated with aging was reported in several studies [53,54,55,56,57,58,59]. However, the underlying mechanism remains to be clarified. One of the possible mechanisms could be lower telomere attrition. To date, the optimum amount of PA and the type of exercise for maintaining TL remains under debate. Of the 83 articles reviewed, 49 analyzed PA and TL [45,46,61,62,63,64,65,66,67,68,69,70,71,72,73,74,75,76,77,78,79,80,81,82,83,84,85,86,87,88,89,90,91,92,93,94,95,96,97,98,99,100,101,102,103,104]. Cross-sectional studies (n = 23) are presented in Table 1 [45,46,61,62,63,64,65,66,67,68,69,70,71,72,73,74,75,76,77,78,79,80,81,82,83,84,85,86,87,88,89,90,91,92,93,94,95,96,97,98,99,100,101,102,103,104]. Table 2 [27,81,82,83,84,85,86,87,88,89,90,91,92,93] shows observational studies (n = 14) using case-control and longitudinal approaches (n = 3); and Table 3 [44,94,95,96,97,98,99,100,101,102,103,104] shows intervention studies. In these tables, the details for each study including author, reference, year, design, participants and characteristics, TL analysis methods, tissue for TL analysis, exposure assessment methods, and main results, are presented.

**Table 1:** Summary of cross-sectional studies on PA and TL.

Author, Reference	Year	Participants	TL Measurements	Tissue for TL Analysis	Exposure Assessment Methods	Results
Cassidy et al. [60]	2010	2284 women from NHS	PCR	Blood	PA assessed by questionnaires	No association was observed between PA and TL
Dankel et al. [61]	2017	4881 subjects from NHANES	PCR	Blood	PA assessed by questionnaires	PA was associated with increased TL, except among those who were overweight or obese
Ding et al. [62]	2018	588 Chinese participants (18–64 y)	PCR	Blood	PA assessed by questionnaires	No associations were found between PA and TL
Du et al. [63]	2012	7813 women (43–70 y) from NHS	PCR	Blood	PA assessed by questionnaires	Women with moderate or vigorous activity had a longer TL than less active
Fretts et al. [64]	2018	2312 American Indian participants from SHFS	PCR	Blood	PA reported by pedometer	Participants in the upper PA quartiles had longer TL
Hastings et al. [65]	2019	6731 participants from NHANES	PCR	Blood	PA determined with different tests (muscle strength, cardiorespiratory capacity (VO_2_) and walking speed	Participants with shorter telomeres performed worse on physical function tests
Kim et al. [66]	2012	44 healthy postmenopausal women	PCR	Blood	PA reported by interview	Regular PA was associated with longer TL
Latifovic et al. [67]	2016	477 subjects (20–50 y)	PCR	Blood	PA assessed by questionnaires	Vigorous PA related to longer TL
Loprinzi [68]	2015	6405 participants from NHANES	PCR	Blood	Sedentary PA on screens (TV, computer …) reported by interview	Sedentary behavior based on screen free time was inversely associated with TL
Loprinzi et al. [69]	2015	6503 participants from NHANES	PCR	Blood	PA reported by interview	Lower TL among those who performed less PA
Loprinzi et al. [70]	2016	6474 participants from NHANES	PCR	Blood	PA reported by interview	The specific PA of running was the only activity associated with TL
Ogawa et al. [45]	2017	6933 participants from NHANES	PCR	Blood	PA reported by interview and VO_2_max in subsample	Those who undertake vigorous PA present longer TL than those who practice moderate PA or do not perform PA
Sassenroth et al. [71]	2015	815 participants from BASII	PCR	Blood	PA assessed by questionnaires	PA was associated with longer TL only when PA was regular and ≥10 y preceding the assessment
Savela et al. [72]	2013	204 participants from HBS	SB	Blood	PA assessed by questionnaires	An U-shaped association was found. Moderate PA associated with longer TL
Shadyab et al. [73]	2017	1476 elderly women from WHI	SB	Blood	PA assessed by questionnaires	Longer TL in the group of more active women compared to those who undertook less PA
Shadyab et al. [74]	2017	1481 elderly women from WHI	SB	Blood	PA assessed by questionnaires and accelerometer	Longer sedentary time was associated with shorter TL in physically inactive older women
Shadyab et al. [75]	2017	1405 elderly women from WHI	SB	Blood	PA assessed by questionnaires and accelerometer	Women at the highest level of total PA compared to the lowest level had longer TL, but significance was lost after adjustment for confounded terms
Silva et al. [76]	2017	Elderly subjects (65–85 y) with intense and moderate training, and never trained	Flow-FISH	Blood	PA assessed by questionnaires and VO_2_max	TL was longer in the intense training group compared to the untrained group
Song et al. [77]	2010	170 subjects (18–80 y)	PCR ELISA	Blood	PA assessed by questionnaires	PA was inversely correlated with biomarkers of DNA damage, and these biomarkers were negatively associated with TL
Tucker LA [46]	2017	5.823 adults from NHANES	PCR	Blood	PA assessed by questionnaires	PA was inversely associated with TL. High levels of PA were associated with longer telomeres, with a biological aging of 9 y difference compared with the sedentary ones
von Känel et al. [78]	2017	African (n = 96) and Caucasian (n = 107) schoolteachers from SAABPAS	PCR	Blood	PA measured by accelerometry	Regular PA of different intensity was not directly associated with TL
Williams et al. [79]	2017	5674 adults from NFBC	PCR	Blood	PA determined by physical tests	TL was associated with greater aerobic fitness and muscular endurance only in young adults
Xue et al. [80]	2017	518 adults from China	SB	Blood	PA assessed by questionnaires	Watching TV was associated with shorter TL However, doing moderate or vigorous PA was not associated with TL

TL: telomere length; bp: base pairs; PA: physical activity; y: years old; TV: television; h: hour; d: day; w: week; m: month; y: year; VO_2_max: maximal oxygen uptake; T2D: type 2 diabetes; ISRT: Individual Shear Rate Therapy; PCR: Polymerase Chain Reaction; SB: Southern blot analysis of terminal restriction fragment lengths (TRF); GE: gene expression; GWEA: genome-wide expression arrays; TERT: telomerase reverse transcriptase gene; WB: Western blotting; TERRA: telomeric repeat-containing RNA gene; FISH: fluorescence in situ hybridization; FACS: fluorescence activated cell sorting; NHS: Nurses’ Health Study; NHANES: National Health and Nutrition Examination Survey; SHFS: Strong Heart Family Study; BASII: Berlin Aging Study II; HBS: Helsinki Businessmen Study; WHI: Women’s Health Initiative Objective Physical Activity and Cardiovascular Health study; SAABPAS: Sympathetic activity and Ambulatory Blood Pressure in Africans study; NFBC: Northern Finland Birth Cohort.

**Table 2:** Summary of case-control (CC) and longitudinal (L) studies on PA and TL.

Author, Reference	Year	Type	Participants	TL Measurements	Tissue for TL Analysis	Exposure Assessment Methods	Results
Colon et al. [81]	2019	CC	7 male triathlon athletes and 7 active controls	PCR	Blood	PA assessed by questionnaires and VO_2_max	The triathlete subjects had longer telomeres than the active controls. Positive association between TL and VO_2_max
Denham et al. [27]	2016	CC	Polish endurance athletes (n = 61) and paired active controls (n = 61)	PCR GE	Blood	PA reported by questionnaires	Endurance athletes have longer TL, as well as a higher expression of TERT mRNA
Denham et al. [82]	2013	CC	67 male ultramarathon runners and 63 controls	PCR	Blood	PA assessed by questionnaires	Ultramarathon runners had telomeres 11% longer than controls
LaRocca et al. [83]	2010	CC	57 participants: differing by age and PA	SB	Blood	PA assessed by questionnaires and VO_2_max	TL of the older endurance-trained adults was greater than their sedentary peers. TL was positively related to VO_2_max
Magi et al. [84]	2018	CC	26 men (20–50 y)	SB	Muscle	PA reported by interview	Regular physical training was positively with the maintenance of TL
Mathur et al. [85]	2013	CC	17 marathon runners and 15 controls	FISH	Blood	Different tests were performed	No differences were observed in the TL of the different PA groups or tests
Muniesa et al. [86]	2017	CC	125 young elite athletes	PCR	Blood	PA reported through a database	Elite athletes had a TL 12.4% longer than controls
Osthus et al. [87]	2012	CC	10 young and 10 elderlies (50% endurance athletes, 50% medium level)	PCR	Muscle	PA assessed by questionnaires and VO_2_max	Older endurance athletes had longer TLs compared to their counterparts. These differences were not seen in the young participants
Puterman et al. [88]	2010	CC	63 postmenopausal women	PCR	Blood	PA assessed by questionnaires	No association between PA and TL was observed. However, vigorous PA was linked to TL
Rae et al. [89]	2010	CC	18 endurance runners vs. 19 controls	SB	Muscle	PA assessed by questionnaires	No significant differences were found between the mean and minimum TL between the endurance runners and controls
Simoes et al. [90]	2017	CC	Elite sprinters (n = 11) and untrained controls	PCR	Blood	PA reported by medical history	Elite sprinters have longer TL than their paired controls
Soares-Miranda et al. [91]	2015	L	582 elderly subjects from CHS	SB	Blood	PA assessed by questionnaires and different tests	At baseline, higher PA was associated with longer TL. Prospective analyses show that changes in PA were associated with differences in changes in TL
Stenbäck et al. [92]	2019	L	700 elderly subjects (Finland)	PCR	Blood	PA assessed by questionnaires and accelerometry	PA for 2-w was not associated with TL after adjustment. However, moderate PA was associated with longest TL
Weischer et al. [93]	2014	L	4576 participants from CCHS	PCR	Blood	PA assessed by questionnaires	Increased PA was associated with short TL at baseline, but not with a change in TL during 10 years of follow-up

TL: telomere length; bp: base pairs; PA: physical activity; y: years old; TV: television; h: hour; d: day; w: week; m: month; y: year; VO_2_max: maximal oxygen uptake; T2D: type 2 diabetes; PCR: Polymerase Chain Reaction; SB: Southern blot analysis of terminal restriction fragment lengths (TRF); GE: gene expression; TERT: telomerase reverse transcriptase gene; WB: Western blotting; CCHS: Copenhagen City Heart Study; HRS: Health and Retirement Study; CHS: Cardiovascular Health Study.

**Table 3:** Summary of intervention studies on PA and TL.

**Author, Reference**	**Year**	**Participants**	**TL Measurements**	**Tissue for TL Analysis**	**Intervention**	**Results**
Borghini et al. [94]	2015	17 male endurance athletes and 32 controls	PCR	Saliva	Ultra-endurance race in athletes (330 km in a maximum time of 150 h)	Endurance athletes present longer TL than controls. Acute extreme intervention increased TL attrition compared to baseline
Brandao et al. [95]	2020	20 obese sedentary women (20–40 y)	PCR	Blood	Combined aerobic and strength training (55 min/d, 3 times/w, 8 w)	Combination training for 8 w promoted an increase in TL, fat-free mass, and physical performance
Chilton et al. [96]	2014	22 healthy young men, nonsmokers	GE	Blood	30 min of continuous running on a treadmill at 80% O_2_	Increased expression of the key telomeric gene TERT mRNA, associated with longer TL
Diman et al. [97]	2016	10 healthy and moderately active young men	FISH GE	Muscle	Two cycling exercise groups: low intensity (50% VO_2_) and a high intensity (75% VO_2_).	Endurance cycling exercise increased the TERRA gene expression, a key player in telomere integrity, associated with longer TL
Dimauro et al. [98]	2017	24 men, 12 T2D and 12 controls.	FISH	Blood	Two groups: untrained and trained (moderate PA over at least 1 y)	Individuals who participated in the regular exercise program showed longer TL
Friedenreich et al. [99]	2018	212 Healthy women aged (50–74 y).	PCR	Blood	Two groups: aerobic exercise (45 min, 5 d/w for y, n = 99) vs. inactivity	No association between aerobic exercise and TL
Laye et al. [100]	2012	8 participants in the “2010 Bornholm Ultramarathon 7”	PCR GE	Muscle and whole blood	Carrying out 7 marathons on 7 consecutive d	A positive regulation of gene expression of the components of the shelterin complex was found without observing changes in TL
Mason C et [101]	2013	439 women with overweight or obesity (50–75 y)	PCR	Blood	Four groups: weight loss with diet (n = 118), PA (n = 117), diet + exercise (n = 117) and control (n = 87); for 12 m	After 12 m, no changes were seen in the TL of the intervention groups compared to the control group
Puterman et al. [102]	2018	68 careers of elderly people	PCR	Blood	Two groups: aerobic training and control for 24 w	Significant changes in TL were observed in aerobic exercise intervention group
Sjögren et al. [103]	2014	49 participants (68 y) with low PA	PCR	Blood	Two groups: PA and minimal PA. Intervention for 6 m	The decrease in seated time in the intervention group was associated with TL
Werner et al. [44]	2019	125 inactive participants	PCR FlowFISH FACS	Blood	Three groups: aerobic endurance training; high intensity exercises; resistance training	Endurance training and high intensity, but not strength training, was associated with increased telomerase activity and TL
Zietzer et al. [104]	2017	26 youth and 14 elderly participants	PCR	Blood	Randomization to 2 trials: -ISRT-1: crossover trial (2 separate sessions). -ISRT-2: 1 session of ISRT, and subsequently 5 d/w of 90 min.	Telomerase activity in young individuals increased with exercise. In contrast, short-term ISRT exercises were not associated with telomerase activity. However, after 5 w of daily ISRT, telomerase increased

TL: telomere length; bp: base pairs; PA: physical activity; y: years old; TV: television; h: hour; d: day; w: week; m: month; y: year; VO_2_max: maximal oxygen uptake; T2D: type 2 diabetes; ISRT: Individual Shear Rate Therapy; PCR: polymerase chain reaction; SB: Southern blot analysis of terminal restriction fragment lengths (TRF); GE: gene expression; TERT: telomerase reverse transcriptase gene; mRNA: micro-ribonucleic acid (RNA fragment); WB: Western blotting; TERRA: telomeric repeat-containing RNA gene;; FISH: fluorescence in situ hybridization.

Most studies on the effect of PA on TL (37/49) were analyzed at the observational level in most cross-sectional studies (Table 1); PA was associated with longer TL. However, this association was mainly reported in subgroup analyses and not for the whole population included in the study. This subgroup analysis involved participant characteristics (men or women, older or young, among others) or different levels of PA (active versus inactive, moderate versus vigorous, regular, or not regular, etc.) Thus, as multiple comparisons were carried out in the same study, some of these comparisons could be statistically significant by chance, if no correction for multiple comparisons was considered. Therefore, the effect of PA on TL in these cross-sectional studies carrying out multiple comparisons is probably overestimated in the published papers. Among the cross-sectional studies, 3 [60,62,78] did not observe any association between PA and TL. The others reported mixed results (no association for some subgroups, but associations in at least one subgroup). One study [72], reported a U-shaped association, with only moderate PA being associated with longer TL. In these cross-sectional studies [45,46,60,61,62,63,64,65,66,67,68,69,70,71,72,73,74,75,76,77,78,79,80], heterogeneity was also detected in the methodology used. PA was assessed by questionnaires in the most studies: some also included objective assessment (accelerometers, pedometers, or several PA tests) in the whole population or in subsamples. Likewise, variability in the methods used to assess TL was detected (17/23 used quantitative PCR, 5/23 used southern blot and 1/23 used Flow-FISH). Blood was the tissue selected for analysis in cross-sectional studies.

To note, several publications (7/24) were carried out on the same epidemiological study [in the United States population of the “National Health and Nutrition Examination Survey” (NHANES)] analyzing different levels of PA or participant characteristics [45,46,61,65,68,69]. Although the results differed depending on the subgroup and exposure analyzed, in general, in NHANES, undertaking any PA is associated with longer TL [45,46,61,62,63,64,65,66,67,68,69]. Thus, individuals that undertook vigorous PA presented greater TL than those with moderate activity or those who did not undertake any PA. No differences were observed between the two latter groups [45]. However, in a subgroup analysis in NHANES, the authors reported that the presence of overweight or obesity could counter the protective effect of PA on TL [61]. Moreover, the differences between the degree of PA and TL were only observed among individuals aged 40 to 64 years [69].

In NHANES, individuals reporting vigorous activity had longer TL (reported in bp increase) (than sedentary individuals and those reporting low or moderate PA. Other studies in different populations, also estimated the effect of PA in terms of bp. Thus, Shadyab et al. [73], in a group of post-menopausal women in the Women’s Health Initiative (WHI) study, observed a significant difference in bp between those who reported greater PA compared to those reporting the least PA. Moreover, a greater TL was observed in women whose walking speed was faster [73]. Similarly, in women from the Nurses’ Health Study (NHS), it was observed that those who undertook vigorous or moderate activity for 2–4 h a week had a greater TL than those less active or sedentary women. Additionally, in the Berlin Aging Study II (BASII), it was estimated that regular PA over the previous 10 years was required to achieve a sustained effect on TL [71].

Furthermore, the role of type of PA, such as easy or fast walking, cycling, or undertaking aerobic or callisthenic exercises, on TL was assessed. Studies note an increase in TL in those performing aerobic or callisthenic exercise only [63]. Nevertheless, these results are contradictory to those found in this same study in a population of lower sample size in previous years [60]. In NHANES, only running was associated with greater TL, this relationship perhaps explaining the benefits of aerobic activity on certain pathologies or on total mortality [70]. Regarding more moderate activities—participants who accumulated more steps or who were in the highest quartile had longer TL than those with fewer steps per day [64]. In longitudinal studies, an increase in exercise-induced caloric expenditure was associated with less shortening of the telomeres over time [73]. Regarding only sedentary individuals in a women’s accelerometer study, a difference of 170 bp was observed between the women in the highest sedentary quartile and those of the lowest level. However, when the self-reported sedentary lifestyle of the participants was studied, this was not associated with TL, possible due to the imprecise nature of self-reported questionnaires [75]. With regard to a sedentary lifestyle in a young adult population, it was observed that adults who spent more time watching the television presented shorter TL than those who watched it less [68]. For each hourly increase of television, the risk of having short telomeres increased by 7% [68]. On classifying the subjects into age groups, this association was only significant in adults from 20 to 40 years old [80].

Table 2 [27,81,82,83,84,85,86,87,88,89,90,91,92,93] shows case-control (n = 11) and longitudinal studies (n = 3) analyzing the association between PA and TL. The results were mixed. In a majority of studies (n = 8/11), TL was longer in physically active cases than in controls. However, in three studies, no differences between TL in cases and control were found [85,88,89]. In general, sample size in the case-control studies was smaller than in the cross-sectional studies and participants characteristics were maximized in terms of PA (for example cases being elite sprinters in comparison with untrained controls [90], or cases being ultramarathon runners in comparison with controls [82]. The methods to assess TL were diverse, including PCR (7/11), Southern blot (3/11) or FISH (1/11). Regarding the type of tissue analyzed, although in general leukocytes were isolated from blood, two studies analyzed muscle [87,89]. Moreover, in one study, gene expression analysis of the Telomerase Reverse Transcriptase gene (TERT) gene was also examined [27]. Specifically, Denham et al. [27] compared several factors associated with cellular aging in endurance athletes with their corresponding active controls and found a greater TL and greater expression of the TERT and TPP1 genes in athletes.

The three prospective studies [91,92,93] also presented mixed results. Weisher et al. [93] found no association between changes in PA and changes in TL over 10 years of follow-up in a large sample size of participants in the Copenhagen City Heart Study (CCHS). However, Soares-Miranda et al. [91] found an association between changes in PA and in TL in subjects participating in the Cardiovascular Health Study (CHS). Subgroup associations were found in the third study [92].

Table 3 [44,94,95,96,97,98,99,100,101,102,103,104] shows intervention studies (n = 12) analyzing the association between PA and TL. Several trials (9/11) found that individuals in active PA interventions had an increase in TL relative to controls, but others did not observe such differences (3/11) or reported subgroup results. Again, mixed results were found, mainly due to the great heterogeneity of the interventions, study designs (non-controlled or controlled, and different control groups among the randomized trials), sample size, as well as the participant’s characteristics. Thus, seven studies measured TL using only PCR; in one study, PCR was used combined with gene expression or Flow-FISH; one study used FISH, and in two, gene expression (combined with microRNA analysis in one of them), was used. Likewise, some heterogeneity was detected in the tissue analyzed (mainly in blood, but two in muscle and one in saliva). All of this contributed to the possible heterogeneity.

Three intervention trials did not report associations between PA and TL [99,100,101], but even these trials were very heterogeneous. Friedenreich et al. [99], analyzing 212 healthy women aged 50–74 years and comparing 1 year intervention (in two groups: a group with aerobic exercise, 45 min/d, 5 d/week versus an inactivity group) did not find significant association between aerobic exercise intervention and TL. Laye et al. [100] did not observe changes in TL by subjecting eight trained individuals to seven marathons in seven consecutive days. However, there was greater mRNA regulation of the shelterin complex, involved in TL protection [100]. A trial of 439 elderly post-menopausal women [101] compared TL after 12 months of PA and/or dietary intervention, plus a control group, and did not find any differences between the different groups. However, after 12 months, in a subgroup analysis, the women with the shortest baseline telomeres had a greater mean increase in TL than those with the longest baseline TL.

An important consideration for intervention trials is the length of the intervention. Brandao et al. [95] observed that an 8-w intervention combining aerobic and strength training in sedentary women with obesity increased TL. However, it has been reported that different training modalities may exert different effects. Thus, Werner et al. [44] in a complex intervention showed that endurance and interval training increased TL, but not endurance training. In this study, telomerase activity was also measured and was positively correlated with TL. Similarly, Zietzer et al. [104] in an intervention study carried out in youth and elderly participants showed that in the youngest population, telomerase activity increased significantly in a single session of aerobic exercise [104]. When endurance athletes were compared with individuals with an average level of activity, a greater TL was observed compared with the control group.

However, on stratifying by age, this difference was not observed in the youngest group (22–27 years) but was much more noticeable in the elderly group (66–77 years) [104].

With regard to the different types of PA, the results have been mixed, mainly due to the heterogeneity of the intervention, tissue, and assessment method and to the small sample sizes. Bicycle endurance exercise has been associated with increased gene expression levels of “Telomeric repeat-containing RNA” (TERRA) that is transcribed from the telomeres and is associated with longer TL, suggesting benefits of aerobic exercise [97]. Likewise, an upregulation of various telomeric genes, such as the TERT or SIRT6 (Sirtuin 6) genes, as well as a differential regulation in microRNA that codifies genes involved in the homeostasis of telomeres was found after 30 min of intense cardio-respiratory exercise in young males [96].

### 3.2. Association between Telomere Length and Tobacco Smoking

Of the 83 articles reviewed, 27 analyzed cigarette smoking and TL, all of which were observational studies (ethical reasons are crucial here explaining the lack of intervention studies). We grouped these observational studies in cross-sectional (Table 4) [60,67,77,93,105,106,107,108,109,110,111,112,113,114,115,116,117,118,119,120] and in case-control and longitudinal studies (Table 5) [121,122,123,124,125,126,127]. There were 20 cross-sectional studies [60,67,77,93,105,106,107,108,109,110,111,112,113,114,115,116,117,118,119,120], 2 case-control studies [124,127], and 5 longitudinal studies [121,122,123,125,126]. Despite some heterogeneity in the results, population characteristics, and methodology used, most studies show an association between tobacco smoking and reduced TL.

Only 2 of the 21 cross-sectional studies report no association between tobacco smoking and TL [60,105]; in one additional study, the initial association between tobacco smoking and TL was non-significant after adjusting for BMI [120]. In another report [108], although smoking status was not associated with TL, there was an inverse association between the number of cigarettes smoked and TL. The initial investigation by the NHS, analyzing the association between smoking and TL in 2284 women, found no relationship between both variables [60]. Two years later, in 5862 women, that same study observed a slight association between TL and smoking, specifically 8% greater TL in subjects who had never smoked as opposed to smokers [117] The authors stated that the combined effect of various risk factors (diet, PA, weight, alcohol, and tobacco) was much greater than the individual additive effect [117]. Various authors have also found greater telomere shortening in smokers as opposed to non-smokers [119,121,122,123]. Tobacco possibly accounts for 0.64–1.23% of TL variation [116]. More specifically, there could exist 50 bp of variation between smokers and non-smokers [119], 73 bp [125], or 190 bp according to others [112].

**Table 4:** Summary of cross-sectional studies on smoking and TL.

Author, Reference	Year	Participants	TL Measurements	Tissue for TL Analysis	Exposure Assessment Methods	Results
Cassidy et al. [60]	2010	2284 women from NHS	PCR	Blood	Tobacco smoking reported by questionnaire	Tobacco use was not associated with TL
Flannagan et al. [105]	2017	20 smokers and 20 non-smokers	PCR	Blood	Tobacco smoking reported by questionnaire	No association was detected between smoking and TL
Gao et al. [106]	2016	548 participants from ESTHER	PCR and methylation	Blood	Tobacco smoking reported by questionnaire	Lower methylation was observed in current smokers associated with TL
Huzen et al. [107]	2014	8592 subjects from PREVEND	PCR	Blood	Tobacco smoking reported by questionnaire	Dose-dependent association between number of cigarettes smoked and baseline TL
Khan et al. [108]	2019	5864 participants from NHANES	PCR	Blood	Tobacco smoking reported by questionnaire	No association was detected between TL and smoker status, but there was an inverse association between the number of cigarettes smoked and shorter TL
Latifovic et al. [67]	2016	477 subjects (20–50 y)	PCR	Blood	Tobacco smoking reported by questionnaire	Smokers had shorter TL than those who had never smoked
Lei et al. [109]	2020	500 African Americans from FACHS	Methylation arrays	Blood	Tobacco smoking reported by questionnaire	Associations between smoking and aging by differences in methylation
Lu et al. [110]	2019	2256 participants from WHI and JHS; 1078 participants from FHS	SB and methylation arrays	Blood	Tobacco smoking reported by questionnaire	Being a smoker was associated with lower values of mtlDNA (level of DNA methylation as an estimator of TL). In addition, smoking was associated with a shorter TL
Lu et al. [111]	2017	1303 non-smoking adult participants from GS:GFHS	PCR	Blood	Tobacco smoking reported by questionnaire	TL decreased more rapidly with increasing age among passive smokers compared to those who were not exposed
Nawrot et al. [112]	2010	216 non-smokers and 89 smokers from FLEMENGHO	SB	Blood	Tobacco smoking reported by questionnaire	The TL of smokers was shorter than in non-smokers
Needham et al. [113]	2013	5360 subjects from NHANES	PCR	Blood	Tobacco smoking reported by questionnaire (number of cigarettes/d and years of smoking)	Smokers of 60 packs of cigarettes/y or more showed significantly shorter TL than those who had never smoked
Patel et al. [114]	2017	461 subjects NHANES	PCR and GE	Blood	Tobacco smoking reported by questionnaire	The number of cigarettes smoked/d was associated with a shorter TL, but no differences were detected in the expression levels of candidate genes
Rode et al. [115]	2014	55,568 participants from CGPS	PCR	Blood	Tobacco smoking reported by questionnaire	An association was observed between high cumulative tobacco use and short TL
Song et al. [77]	2010	170 adults (18–80 y)	PCR	Blood	Tobacco smoking reported by questionnaire	Inverse association between smoking and TL
Sulastri et al. [116]	2017	130 Minangkabau men (40–50 y)	PCR	Blood	Tobacco smoking reported by questionnaire	Smoking was a risk factor for telomere shortening
Sun et al. [117]	2012	5862 women from NHS	PCR	Blood	Tobacco smoking reported by questionnaire	Compared to current smokers, women who had never smoked had longer TLs
Verde et al. [118]	2015	147 healthy smokers from Spain	PCR	Blood	Smoking habit was determined by survey and Fagerström test	Association between cumulative tobacco use and years of smoking and reduction in TL. No significant differences between the values of metabolized nicotine and TL
Weischer et al. [93]	2014	4576 participants from CCHS	PCR	Blood	Tobacco smoking reported by questionnaire	Short TL was associated with increased tobacco use
Wulaningsih et al. [119]	2016	6456 participants from NHANES	PCR	Blood	Questionnaires for smoking and serum cotinine levels were evaluated	Being a smoker was associated with a 50 bp decrease in TL compared to those who had never smoked
Yun et al. [120]	2019	1037 adults (729 white and 308 African American)	SB	Blood	Tobacco smoking reported by questionnaire	An association was observed between smoking and TL. Body weight had a suppressing effect on this association

TL: telomere length; PCR: polymerase chain reaction; SB: Southern blot analysis of terminal restriction fragment lengths (TRF); GE: gene expression; NHS: Nurses’ Health Study; ESTHER: Epidemiologische Studie zu Chancen der Verhütung, Früherkennung und optimierten Therapie chronischer Erkrankungen in der älteren Bevölkerung (German); PREVEND: Prevention of Renal and Vascular End-Stage Disease study; NHANES: National Health and Nutrition Examination Survey; y: year; FACHS: Family and Community Health Study; WHI: Women’s Health Initiative Objective Physical Activity and Cardio-vascular Health study; JHS: Jackson Heart Study; GFHS: Scottish Family Health Study; FLEMENGHO: Flemish Study on Environment, Genes and Health Outcomes; CGPS: Copenhagen General Population Study; CCHS: Copenhagen City Heart Study; HRS: Health and Retirement Study.

Ex-smokers have a lower loss of TL compared to current smokers [119]. Similarly, a significant inverse relationship was found between the number of cigarettes consumed and TL [108,114].

Regarding longitudinal studies (Table 5) [121,122,123,125,126], more discrepancies were found. Although in these studies, significant associations between tobacco smoking and TL were found at baseline, some of these studies, such as the Epidemiologische Studie zu Chancen der Verhütung, Früherkennung und optimierten Therapie chronischer Erkrankungen in der älteren Bevölkerung (ESTHER) cohort [125], did not detect longitudinal associations. However, other longitudinal studies [122,123] reported significant associations in the follow-up. In order to explain the potential discrepancies in cross-sectional and longitudinal associations, Zhang et al. [121] analyzed in 5624 participants from an American population. They analyzed differences in TL between smokers and non-smokers following 16 years of follow-up, with concurrent and past smoking status reported biennially, and taking into account the sex influence. The authors observed that smoking was associated with reduction in TL over time, but this association was attenuated in men. The reason for this attenuation was a higher rate of smoking cessation among men with shorter TL, probably because of the poor health of these individual with shorter TL. In conclusion, this study suggested that time-varying and sex-specific associations should be measured in the studies analyzing the association between tobacco smoking and TL.

As with PA, several methodologies were used in studying the association between tobacco smoking and TL. The most frequent method was by PCR, followed by southern blot. However, other techniques were also used. It is interesting to mention the assessment of DNA methylation in several studies [106,109,110], taking into account the strong association between tobacco smoking and DNA methylation.

**Table 5:** Summary of case-control and longitudinal studies on smoking and TL.

Author, Reference	Year	Type of Study	Participants	TL Measurements	Tissue for TL Analysis	Exposure Assessment Methods	Results
Zhang et al. [121]	2016	Longitudinal	5624 participants from HRS, 16 y follow up	PCR	Saliva	Tobacco smoking reported by questionnaire	Shorter TL observed in smokers. The number of cigarettes was also inversely associated with TL in women, but not in men. Sex-specific prospective changes on smokers and TL were detected
Bendix et al. [122]	2014	Longitudinal	1356 individuals (30–70 y). 10-y follow-up	PCR	Blood	Tobacco smoking reported by questionnaire	Tobacco influences the change in TL after follow-up, with smokers having a greater risk of telomere shortening
Chen et al. [123]	2011	Longitudinal	Follow-up of 271 participants at 3 times	SB	Blood	Tobacco smoking reported by survey	Smoking at baseline was associated with shorter TL and accelerated shortening
Marcon et al. [124]	2017	Twin study (case-control)	22 homozygous twins (one smoker and the other non-smoker)	SB and methylation arrays	Blood	Tobacco smoking reported by questionnaire, and urine cotinine	Statistically significant higher values of TL in smokers compared to non-smoking twins
Müezzinler et al. [125]	2015	Longitudinal	3600 elderly adults. Follow-up on 1000 participants	PCR	Blood	Tobacco smoking reported by questionnaire	Smoking was inversely associated with TL at baseline. On average, current smokers had 73 bp shorter telomeres, but this relationship could not be shown longitudinally
Strandberg et al. [126]	2011	Longitudinal	622 participants from Finland (30–45 y). Follow-up at 30–40 years	SB	Blood	Tobacco smoking reported by questionnaire	Age-adjusted TL was significantly longer among lifelong non-smokers compared to past or present smokers
Walters et al. [127]	2014	Case-Control	Healthy smokers (n = 29) vs. non-smokers (n = 29)	SB	Epithelial cells or whole blood	Tobacco smoking reported by questionnaire, and urine cotinine	No differences were observed between TL and being a smoker or not

TL: telomere length; y: years; bp: base pairs; PCR: polymerase chain reaction; SB: Southern blot analysis of terminal restriction fragment lengths (TRF); HRS: Health and Retirement Study; CHS: Cardiovascular Health Study.

In addition, some protein biomarkers and gene expression assays have been assessed in various studies (Table 4 and Table 5). For example, Song et al. identifying a higher level of different protein biomarkers of DNA damage and dysfunction of the telomeres in smokers compared to non-smokers [77]. Patel et al. could not find any differences on the levels of candidate gene expression between smokers and non-smokers [114]. On the other hand, contradictory findings were reported in a methylation and gene expression study carried out on twins. The authors observed greater telomerase activity and greater TL in smokers than in non-smokers [124].

Lastly, the effect of second-hand smoke or “passive smoking” and TL has been analyzed and it was suggested that high degree of exposure to second-hand smoke could accelerate the shortening of the telomeres that naturally occurs with aging [111]. However, another publication did not find any association between passive smoking and TL [119]. More research is needed to evaluate the effects of second-hand smoke on TL that takes into account various confounders such as air pollution, environmental work pollutants, and other related factors.

### 3.3. Association between Telomere Length and Sleep

In this systematic review, we analyzed 11 articles on the association between TL and sleep (Table 6) [43,128,129,130,131,132,133,134,135,136,137]. In comparison with the other considered lifestyle factors in this review (PA and tobacco smoking), very few studies focused on sleep. The influence of sleep quality and sleep disorders on aging and on physical and mental health has been recognized more recently [138,139]. In general, aging is associated with poor sleep. Sleep quantity and efficiency vary across the lifespan, sleep duration being shorter, and sleep efficiency being lower at older ages; these difficulties worsen after 60 years, and with some differences between men and women [139,140,141]. Sex-specific differences are complex and vary by outcome [140]. A recent review including 1.1 million people from the Netherlands, United Kingdom and United States, noted that women aged 41 years and older, despite self-reporting shorter sleep duration and lower sleep efficiency than men, had longer and more efficient sleep objectively measured by actigraphy [142], suggesting that the studies analyzing the association between sleep and TL should consider this heterogeneity. Currently there is a great scarcity of intervention trials on this issue.

All but one study identified for this review were observational (Table 6). Moreover, data from the intervention study only analyzed associations between sleep and TL cross-sectionally at baseline [132]. Only one study [135] had longitudinal data. Ten articles measured TL using PCR and 1/11 used southern blot [129]. Similarly, most studies have relied on self-reported sleep characteristics, with only two studies assessing objective data (polysomnography, actigraphy, etc.) [131,134], contributing to a low evidence level. Furthermore, even when self-reported sleep characteristics were assessed by questionnaires, high heterogeneity was observed. In several studies sleep quality and duration was assessed using the Pittsburgh Sleep Quality Index (PSQI). The PSQI [143], assesses sleep habits with a scale including 19 items used to derive seven component scores (sleep quality, sleep latency, sleep duration, habitual sleep efficiency, sleep disturbance, sleep medication and daytime dysfunction. These scores are summed to yield a “global sleep quality score”. Some used the global sleep quality score [132] to analyze the association between sleep quality and TL, while others [128] used the sleep quality sub-scale score. More heterogeneity was observed in other studies analyzing the association between the self-reported sleep quality and TL in which the PSQI was not administered [43,129,130,131,135,136,137].

Another variable commonly investigated was sleep duration. In general, this sleep characteristic was obtained by asking participants to state their average nightly sleep duration. Studies differed in their inclusion of weekend nights and in the response categories used. Some studies also analyzed the influence of insomnia or sleep disturbances on TL [129,135,136] using various questionnaires. Despite this heterogeneity, several studies found a significant association between poorer sleep quality (or sleep duration) and shorter TL [43,128,129,130,135,137]. However, several of these studies observed subgroup-specific effects. For example, Jackowska et al. [130], found an association between sleep duration and TL in men but not in women. Grieshober et al. [129] noted that the association found between longer sleep duration and longer TL in 3145 postmenopausal women from the WHIS cohort was stronger in African American than White women, and did not reach the statistical significance in White individuals when the analysis was stratified by race. Likewise, Liang et al. [43], observed a significant positive association between sleep duration and TL in the NHS. This study further uncovered a modifying effect of age, such that sleep duration was only associated with TL in women younger than 50. Differences by age were also observed by Cribbet et al., [128], but in the opposite direction.

**Table 6:** Summary of studies on sleep and TL.

Author, Reference	Year	Type of Study	Participants	TL Measurements	Tissue for TL Analysis	Exposure Assessment Methods	Results
Cribbet et al. [128]	2014	Cross-sectional	154 subjects (45–77 y)	PCR	Blood	Sleep quality and duration by questionnaire (PSQI)	Better quality of sleep was significantly associated with longer TL. Sleep durations of >7 h/night or ≤5 h/night associated with shorter TL in older adults. Sleep duration in middle-aged adults was not associated with TL
Grieshober et al. [129]	2019	Cross-sectional	3145 post-menopausal women (EA, AA) from the WHIS	SB	Blood	Sleep duration and sleep disturbance by questionnaires (WHIIRS)	Longer TL was associated with longer sleep duration in the whole sample (associations being strongest among AA). No associations were observed with sleep disturbances
Jackowska et al. [130]	2012	Cross-sectional	494 men and women (mean age 63.3 y) from the WIIS	PCR	Blood	Sleep duration assessed by interview	Sleep duration was associated with TL in men, but not in women. TL was 6% shorter in men who sleep ≤5 h/night compared to >7 h/night
Liang et al. [43]	2011	Cross-sectional	4117 women from the NHS	PCR	Blood	Sleep duration and rotating night shifts assessed by questionnaires	Sleep duration was associated with TL (compared to women in the 9 h/night, those in the <6 h/night category, had decreased TL). However, heterogeneity by age was observed (sleep duration and TL were only associated in women <50 y, and not older). No significant associations were found between rotating shift history and TL
Nguyen et al. [131]	2020	Cross-sectional	1070 parents/careers with a (mean age 44 y), from the LSAC	PCR	Blood	Sleep behavior determined by interview and actigraphy over 8 d	Sleep duration and most other sleep metrics were not associated with TL
Prather et al. [132]	2015	Cross-sectional	87 obese adults from a trial (San Francisco, CA) at baseline	PCR (DTC)	Blood	Sleep quality and duration by questionnaire (PSQI)	Poorer global sleep quality was associated with shorter TL in lymphocytes, but not in granulocytes. Sleep duration was not related to TL
Prather et al. [133]	2011	Cross-sectional	245 women (49 to 66 y)	PCR	Blood	Sleep quality and duration by questionnaire (adapted from the PSQI)	Lower sleep quality was associated to shorter TL
Tempaku et al. [134]	2018	Cross-sectional	925 participants from EPISONO cohort	PCR	Blood	Sleep quality and duration assessed by questionnaires (PSQI, UNIFESP, Epworth, ISI, PSPDN), and polysomnography	Insomnia disorders and long sleepers were associated with shorter TL
Wynchank et al. [135]	2019	Longitudinal	2936 European subjects from the NSDA analyzed at two waves 6 y apart	PCR	Blood	Sleep duration and insomnia symptoms by questionnaire (ISR). Chronotype determined by the MCTQ	Indicators of delayed circadian rhythm, Late MSFsc, late sleep onset time, and moderately late chronotype, were associated with shorter TL at both waves. No predictors showed accelerated TL attrition over 6 y. Sleep duration and insomnia were not associated with TL
Zgheib et al. [136]	2018	Cross-sectional	497 Lebanese (men and women >18 y)	PCR	Blood	Sleep habits determined by questionnaire (3 questions)	Difficulties to sleep (but not short sleep duration) were associated with shorter TL
Zhao H [137]	2017	Cross-sectional	12,178 Mexican Americans (20–85 y, 80% women) from the MM:MACS	PCR	Blood	Sleep duration determined by questionnaire	TL was associated with sleeping time per d (longer in those participants who slept at least 9 h/d, followed by those who slept between 7–8 h/d, and shortest in those who slept ≤6 h/d)

TL: telomere length; bp: base pairs; y: years old; h: hour; d: day; y: year; PCR: Polymerase Chain Reaction; PSQI: Pittsburgh Sleep Quality Index; EA: European-American; AA. African-American; WHI: Women’s Health Initiative Objective Physical Activity and Cardio-vascular Health study; WHIIRS: “Women’s Health Initiative” Insomnia Rating Scale; WIIS: Whitehall II study; NHS: Nurses’ Health Study; LSAC: Longitudinal Study of Australian Children; EPISONO: São Paulo Epidemiologic Sleep Study; DTC: PCR in different type of cells; UNIFESP: “Universidade Federal De São Paulo” (UNIFESP) Sleep Questionnaire; Epworth: Epworth Sleepiness Scale; ISI: Insomnia Severity Index; PSPDN: Pre-Sleep–Previous Day and Night; NSDA: Netherlands Study of Depression and Anxiety; ISR: Insomnia Rating Scale; MCTQ: Munich Chronotype Questionnaire; MSFsc: Lack of sleep on days off; MM:MACS: Mano a Mano: Mexican American Cohort Study.

In this population [128], sleep duration was associated with TL only in older, but not in middle-aged, adults. In addition, mixed results were reported on the effect of long sleep on TL (U-shaped or linear effect). For example, Zhao et al. [137], observed longer TL in Mexican Americans who reported sleeping at least 9 h/night, whereas Tempaku et al. [134] in Brazil, observed associations between longer sleep and shorter TL. Cribbet et al. [128] also described a U-shaped association between sleep duration and TL, with the extreme of sleep duration having the shortest TL. Interestingly, in one study examining TL in different types of leukocytes [132], poorer global sleep quality was associated with shorter TL in lymphocytes, but not in granulocytes. This is the only study analyzing the sleep influence in different cell types and additional studies are needed to better understand this relationship. Furthermore, Nguyen et al. [131], using objective measures of sleep (actigraphy), did not find any association between sleep duration, and most other sleep metrics, and TL.

Only one study was identified that assessed the relation between chronotype and TL [135]. Individuals with an evening chronotype had a shorter TL. However, when longitudinal analyses were performed, chronotype did not predict changes in TL. Overall, despite many observational studies reported associations between self-reported sleep duration and sleep quality and TL, these results are mainly cross-sectional, limiting the ability to draw causal inference. More longitudinal and intervention studies are needed to increase our understanding on how poor sleep characteristics affects TL.

## 4. Discussion

This systematic review evaluated the current evidence on the influence of three relevant lifestyle factors (PA, tobacco smoking and sleep characteristics) on TL in adults. In the selected studies on TL, PA was the most studied lifestyle factor, followed by smoking and sleep. In general, being physical active, not smoking and having good sleep quality are associated with a reduced risk of morbimortality for the main chronic diseases [8,9,10,11,144,145,146,147]. There is a challenge in searching for the best biomarker of aging and despite these limitations and the advantage of measuring a combination of biomarkers instead of only one [148,149], TL is the most extensively studied biomarker of age-related diseases [7,22,23,24,25,26,27,28,29,30,31,32,33,34,35,36,37,38,39,40,41,42,43]. In this regard, many results have been obtained focusing on the association between TL, aging, cardiovascular diseases, diabetes, obesity and mortality [35,36,37,38,39,40,148,149]. Therefore, the demonstration of an effect of these lifestyle factors on TL could provide important information on the mechanisms by which these factors have protective health effects, as well as providing evidence to validate TL as a good biomarker of aging with a good capacity to capture healthy lifestyle interventions based on these factors.

However, we have detected that the evidence level of the results obtained on the association among these lifestyle factors and TL is low, these being mainly based on cross-sectional studies. Thus, in this review, no intervention studies were detected analyzing the effect of sleep characteristics on TL. Likewise, due to ethical limitations, no experimental publications have analyzed the effects of tobacco smoking on TL on humans. Regarding PA, there are several intervention studies (12/49) examining the effect of different PA intervention on TL. However, the complexity of the PA interventions hinders comparison between publications and precludes definitive conclusions. Moreover, there is a scarcity of longitudinal studies for all three lifestyle factors. In addition, most of the articles have relied on the self-reported measurement of PA, smoking or sleep parameters, instead of using objective determinations (accelerometers, plasma/urine cotinine, actigraphy, respectively, among others). Moreover, substantial heterogeneity existed across the review studies for several study parameters including sample size and statistical power, participant characteristics (sex, age-group, ethnicity, etc.); biological sample and method used to analyze TL (although in general TL was measured by PCR in leukocytes isolated from blood); and statistical control for potential confounders (adjustment for anthropometric and other lifestyle factors). All of this may contribute to the variations in observed results.

Despite the low-level of scientific evidence provided by the cross-sectional studies analyzed for the three lifestyle factors, and the fact that we cannot discuss the associations in terms of causality, in general, most studies [43,44,46,60,61,62,63,64,65,66,67,68,69,70,71,72,73,74,75,76,77,78,79,80,81,82,83,84,85,86,87,88,89,90,91,92,93,94,95,96,97,98,99,100,101,102,103,104,105,106,107,108,109,110,111,112,113,114,115,116,117,118,119,120,121,122,123,124,125,126,127,128,129,130,131,132,133,134,135,136,137] have reported (for the whole population or at the subgroup level) that physically active individuals have longer TL; that tobacco smoking is associated with shorter TL; and that a poor sleep quality and duration were related to shorter TL. Some studies have postulated several mechanisms by which PA may have an effect on TL. It is speculated that exercise can induce different signaling mechanisms, either epigenetic or transcriptomic [69,150]. Chilton et al. [151], in a comprehensive review on the relationship between exercise, aging and telomeres in human and animal models discussed the lack of good evidence at the mechanistic level to support this association, but suggested reductions in oxidative stress and inflammation as potential mediators. As noted in this systematic review, the optimum amount and the type of PA for maintaining TL remains under debate. Lack of agreement among studies could result from different factors, such as: time—need for interventions longer than 12 months to see the long-term effect of PA on telomeres; variability in trials—major random error and consequent lower precision in estimating the effect and low statistical power in the intervention studies due to the complexity of the intervention; or specific characteristics of the participant that could hinder the protective effect of exercise on telomeres [99]. In addition, a U-shaped relationship is thought to exist between endurance exercise and the regenerative capacity of skeletal muscle [72,89]. The possible negative effect on TL of high intensity exercise could be due to an inducing oxidative stress through an increase in reactive oxygen metabolites and free radicals [150].

Tobacco is one of the main risk factors for cardiovascular events and mortality. In addition, those who start smoking at an early age and continue during adulthood lose a decade of life expectancy compared to never smokers [152]. Tobacco smoking is a source of free radicals and oxidants that increase inflammatory response and oxidative stress [111,119,153]. Both processes could increase telomere shortening, contributing to the acceleration of aging and possibly an increased risk of age-related diseases [115,119,120,125]. Although several cross-sectional studies have reported an inverse association between tobacco smoking and TL, evidence for the causality of this association is low and several confounding factors may play a role.

Finally, interest in analyzing sleep and health status has increased in recent years. The relation between short and long sleep with numerous cardiometabolic pathologies has been studied, and a U-shaped association has been demonstrated [154,155]. However, some inconclusive results exist due to uncertainties in self-reported sleep characteristics, different methodological designs and the different populations analyzed [154,155,156]. Furthermore, the sleep process is highly affected during aging, and elderly individuals suffer the greatest disturbances in their sleep patterns [157]. Several authors claim that aging or the presence of other comorbidities could be the cause of the association found between sleep and TL [131,136]. Our systematic review suggests that there is a possible association between shorter TL and various sleep-related parameters, but without establishing a causal relationship between the two factors. Therefore, more longitudinal and intervention studies on sleep and TL are needed. The methodological differences in the evaluation of sleep and TL increase the inconsistency in the results obtained.

In general, a great number of studies analyzed for PA, smoking and sleep reported statistically significant results in subgroup analysis. These associations may be false positive associations due to multiple comparisons or real associations based on heterogeneous effects such as the previously described differences per sex [158] or per age groups [139]. Furthermore, the beneficial effect of PA, non-smoking, or quality sleep on TL may be due to the interaction between various lifestyle factors, such as those studied in this systematic review, or even with others (diet, educational level, etc.), and more studies are needed to assess the role of healthy lifestyle factor combinations in diverse populations.

## 5. Conclusions

The results obtained in this systematic review show that the influence of PA, smoking, and sleep on TL of adults is mixed, and depends on the type of study, population analyzed, and methodology (study design, exposure and TL assessment, subgroup analysis, etc.). The evidence level of the associations is very low due to the cross-sectional nature of the studies. Future research using prospective and well-powered intervention trials with standardized protocols and objective measures is needed. Despite these limitations, current studies suggest a low-evidence level association between moderate, regular, and daily non-sedentary activities and longer TL. Likewise, not smoking and having optimal sleep duration (7 h) and quality, are associated with longer TL. Further research is needed to improve design and reduce heterogeneity and to consider the diversity by sex, age, ethnic groups, and other lifestyle characteristics.
